# Approach to Localized Squamous Cell Cancer of the Esophagus

**DOI:** 10.1007/s11864-022-01003-w

**Published:** 2022-08-31

**Authors:** Chloe Weidenbaum, Michael K. Gibson

**Affiliations:** 1 University of Tennessee Health Science Center Nashville, Nashville, TN USA; 2 Division of Hematology-Oncology, Department of Medicine, Vanderbilt University Medical Center, Nashville, TN USA; 3 Vanderbilt-Ingram Cancer Center, 2220 Pierce Avenue, Nashville, TN 37232 USA

**Keywords:** Esophageal squamous cell carcinoma, Esophageal cancer, Squamous cell carcinoma, Esophageal review

## Abstract

Esophageal cancer is a leading cause of cancer deaths worldwide, with an increasing incidence in recent decades. The majority of esophageal cancers are squamous cell carcinoma. The 5-year survival rate of esophageal squamous cell carcinoma (ESCC) is poor, and there remains globally a pressing need for novel treatments that improve patient outcomes and quality of life. In this review, we discuss management of localized ESCC with an update on relevant newly published literature, including targeted therapy and novel biomarkers. The standard treatment approach for locally advanced, resectable ESCC is currently chemoradiation with or without surgery. Here we discuss different approaches to endoscopic resection, surgery, and radiation therapy. Although the typical chemotherapy regimen is a combination of a platinum with a fluoropyrimidine or paclitaxel, different regimens are being evaluated. With the landscape of immunotherapy rapidly evolving, at the forefront of new treatments for ESCC is immunotherapy and other targeted agents. Ultimately, the treatment approach should be individualized to each patient.

## Introduction

Esophageal cancer is a leading cause of cancer deaths worldwide, with an increasing incidence in recent decades [1]. In 2020 there were 604,000 new cases and 544,000 related deaths, making it the seventh-most common cancer and sixth-most common cause of cancer-related death globally [2]. Approximately 90% of esophageal cancers worldwide are squamous cell carcinoma (ESCC) [3]. Prevalence and risk factors for ESCC are distinct from that of esophageal adenocarcinoma (EAC) and vary by geographic region, environmental exposures, and patient intrinsic factors. The highest prevalence of ESCC is found in the esophageal cancer belt, which includes parts of northern Iran, central Asia, and China. EAC is predominant in North America and Western Europe [4–6]. While our understanding of risk factors is evolving, the strongest risk factors for ESCC in the esophageal cancer belt are low intake of fruits and vegetables, drinking high-temperature beverages, and higher age. In North America and Western Europe, however, the strongest risk factors are alcohol consumption and smoking [3, 7, 8].

ESCC arises in the upper and mid esophagus, whereas EAC develops in the distal esophagus and gastroesophageal (GE) junction [9, 10]. Up to 10% of patients are asymptomatic at diagnosis. Most patients present with dysphagia, unintentional weight loss, and pain [11–13]. The gold standard for diagnosis is esophagogastroduodenoscopy (EGD) and biopsy [14]. Local staging is most accurate with endoscopic ultrasound (EUS), while cross-sectional imaging with CT, PET scanning (preferred), or MRI evaluates for distant disease [15]. Treatment options and prognosis are largely dependent on the American Joint Committee on Cancer Tumor, Node, Metastasis (TNM) staging system [16]. Locally advanced disease is treated for cure with multi-modality therapy, while advanced disease is treated with palliative intent.

Despite improvement in patient outcomes over the past few decades, as of 2016 the 5-year survival (for ESCC and EAC combined) remains relatively poor, at 47% for localized disease and 25% for regional disease [17]. There remains globally a pressing need in ESCC for novel treatments that improve patient outcomes and quality of life (QOL). In this review, we discuss management of localized ESCC with an update on relevant newly published literature.

## Methods

We searched new publications on PubMed from approximately the past year, 9/1/2020–9/1/2021. The keywords “esophageal squamous cell carcinoma” were used, and relevant publications were included.

## Epidemiology — association with HPV

Petrelli and colleagues published a systematic review and meta-analysis that explored the association between ESCC and human papillomavirus (HPV) types 16 and 18. Over 100 studies have investigated the relationship between HPV and ESCC more broadly. This is a review and meta-analysis involving 145 prospective and retrospective studies that evaluated the incidence, risk, and prognosis of HPV-16/18-related ESCCs in 16,484 adults. Limitations of this study included patients being primarily from Asia, methods of HPV detection being DNA FISH only, and lack of adjustment for covariates (such as smoking status). Although it is unclear whether this is etiologic, HPV infection was associated with increased risk of having ESCC (OR = 3.81; 95% CI 2.84–5.11; *P* < 0.001). This suggests a moderate but significant association of HPV-16/18 with ESCC, with HPV-16/18 being detectable in 1 of 5 cases of ESCC albeit with varying prevalence across the world [18].

## Diagnostic modalities

Despite the use of EUS, CT, and PET, some reports show that staging accuracy remains low for sensitivity of nodal involvement. Lee et al. conducted a review and meta-analysis to evaluate the diagnostic performance of MRI for staging of ESCC and EAC. Twenty studies involving 984 patients were included, about 70% with ESCC. Pooled accuracy for stage T0 versus stage T1 or higher had a sensitivity of 92% (95% CI: 82–96) and a specificity of 67% (95% CI: 51–81). Pooled accuracy for stage T2 or lower versus stage T3 or higher had a sensitivity of 86% (95% CI: 76–92) and a specificity of 86% (95% CI: 75–93). Pooled accuracy for stage N0 versus stage N1 or higher had a sensitivity of 71% (95% CI: 60–80) and a specificity of 72% (95% CI: 64–79). This suggests that MRI has high sensitivity but low specificity for T and N staging of esophageal carcinoma. Four of these studies directly compared MRI to CT and PET and found no significant difference in detection accuracy. However, sensitivity rates of MRI were superior to previously found sensitivity rates of CT and PET. The authors suggest that due to its high sensitivity, MRI may be best used for detecting locally advanced disease and surveillance after neoadjuvant therapy [19].

## Endoscopic staging and treatment

Chemoradiation (CRT) +/- surgery is the standard treatment approach for locally advanced, resectable ESCC. For patients with T1N0 ESCC, surgery, endoscopic resection (by ESD or EMR), or radiation therapy (RT) alone are options, and the treatment approach should be individualized to each patient [20–22]. The choice of endoscopic submucosal dissection (ESD) versus endoscopic mucosal resection (EMR) is controversial. Wang et al. conducted a meta-analysis that reviewed their safety and efficacy. A total of 1462 patients with 1650 lesions from 9 studies were included. The study did not indicate how many patients had ESCC versus EAC. En bloc resection rates were 67.94% with ESD versus 52.78% with EMR (OR = 19.79, *p* = 0.000). Complete resection rates were 75.57% with ESD versus 59.47% with EMR (OR = 16.10, *p* = 0.000). The local recurrence rate was also lower in the ESD group, indicating that ESD is superior to EMR in the treatment of esophageal cancer [23].

A systematic review and meta-analysis conducted by Han and Sun similarly evaluated 22 studies for the safety and efficacy of ESD versus EMR for the treatment of superficial esophageal carcinoma (presumably T1, although the study does not specific exact staging). Twelve of the 22 studies involved patients with SCC. Higher en bloc (OR = 43.93; 95% CI 17.16–122.44; *P* < 0.0001), curative (OR = 14.54; 95% CI 5.09–41.54; *P* < 0.0001), and R0 resection rates, and lower recurrence (OR = 0.09; 95% CI 0.04–0.19; *P* < 0.0001) rates were seen with ESD, but only when the lesion size was greater than 20 mm. ESD was significantly more time-consuming and induced more perforations than EMR. These results are significant for ESCC. The authors concluded that ESD might be preferable for lesions above 20 mm, EMR for lesions less than or equal to 10 mm, and both for lesions between 11 and 20 mm [24].

Despite the lower recurrence rate demonstrated with ESD compared to EMR, the risk of recurrence of small lesions is measurable. One randomized trial evaluated ESD followed by (RT) as a method to reduce recurrence of early stage ESCC (T1aN0M0). Seventy patients were randomized to RT or observation following ESD. The median radiation dose was 59.4 Gy completed within 2 months after ESD. No patients had lymph node or distant recurrence, none died, and no severe RT toxicities were reported. Three patients in the non-RT group had mucosal recurrences, all of whom underwent a second ESD. Thus, the 3-year cumulative recurrence-free survival (RFS) was 100% in the radiotherapy group and 85.3% in the non-radiotherapy group (*P* = 0.04; hazard ratio 0.08, 95% confidence interval [CI] 0.01–0.86). Within the T1a invasion subgroups analyzed (T1a extending to the epithelial layer, proper mucosal layer, and muscularis mucosa layer), there was no significant difference in RFS (*P* > 0.05). RT following ESD may be a safe and effective treatment to prevent recurrence of T1a ESCC [25].

The effectiveness of endoscopic treatment remains limited by the potential presence of nodal disease, even when EUS does not detect it. Studies of the risk/predictive factors for lymph node metastasis (LNM) in superficial (T1) ESCC aim to guide the choice between endoscopic treatment and esophagectomy. Xu et al. conducted a review and meta-analysis of 20 studies of 3983 patients to evaluate the risk factors for LNM in stage T1 ESCC in Asian patients undergoing esophagectomy. Tumor size, macroscopic type of tumor (protruding and depressed type as opposed to flat type), degree of differentiation, depth of invasion, and lympho-vascular involvement were risk factors for LNM in T1 ESCC. Age, sex, and tumor location were not. Knowledge of these risk factors can guide the clinician in assessing a patient’s risk of LNM — if low risk, there is a greater chance endoscopic treatment will be sufficient, as LN dissection would not be needed. If higher risk for LNM, esophagectomy and radical lymphadenectomy would be more appropriate [26].

Jiang et al. conducted a similar systemic review and meta-analysis that included 17 studies and 3775 patients with T1 ESCC. Tumor size (over 2 cm), macroscopic tumor type (non-flat type), depth of tumor invasion (T1b stage), poor differentiation, and lympho-vascular involvement were significantly associated with LNM. In contrast to the study performed by Xu et al., Jiang et al. additionally found tumor location (lower esophagus) to be associated with LNM [27]. Of the 2291 patients for which tumor location was studied, 719 (31%) had lower esophageal tumors. One hundred seventy-eight of these patients (25%) experienced LNM. This is compared to 1572 patients who had upper-middle esophageal tumors, 315 (20%) of whom experienced LNM. Although most ESCC is upper or mid-esophagus, those occurring in the lower esophagus were associated with a higher risk of LNM. This may be due to the lymphatic drainage system. During embryogenesis, the middle esophagus and lower esophagus stretch as lymphatic networks develop in the submucosal layer, allowing skip LNM to later develop [28].

The preSANO trial (ESCC and EAC) compared the presence of tumor in the pathologic specimen versus endoscopic biopsies obtained after CRT in order to assess the predictive value of these biopsies to detect pathologic residual disease. Resection specimens and endoscopic biopsies were reviewed independently by two GI pathologists. One hundred nineteen patients were enrolled. Residual tumor was present in the endoscopic biopsies in 70 patients, confirmed on histological analysis of the resected specimen. Nine of these patients had ESCC versus 49 with EAC. Residual tumor was present in the resection specimen from 27 of the other 49 patients despite endoscopic biopsies being negative, making the negative predictive value of endoscopic biopsy low (see Table 1). Of these 27 patients, residual tumor was located in the mucosa in 18 and in the submucosa beneath tumor-free mucosa in 8. Thus, most of the residual disease after nCRT missed by endoscopic biopsies was located in the mucosa. More sampling, sampling larger areas of esophageal mucosa, and considering submucosal biopsies could potentially improve active surveillance after nCRT with surgery [29].

**Table 1:** Sensitivity, specificity, PPV, and NPV of endoscopic biopsy versus surgical specimen

	Endoscopic biopsy	Surgical specimen
Sensitivity	72%	100%
Specificity	100%	100%
Positive predictive value (PPV)	100%	100%
Negative predictive value (NPV)	45%	100%

## Surgery

To study an alternative approach to conventional esophagectomy, Rezai et al. examined the use of a combined mediastinoscopic approach (video mediastinoscope-assisted esophagolysis with trans-hiatal esophagectomy) versus conventional trans-hiatal esophagectomy in patients with esophageal cancer, primarily ESCC (58 out of 62). Patients in the first group underwent trans-hiatal esophagectomy, while patients in the experimental group underwent release of the thoracic esophagus through a neck incision (mediastinal esophagolysis) using a mediastinoscope as part of the trans-hiatal approach. The mean volume of blood loss during the operation, mean operative time, intensive care unit stay, cardiopulmonary complications, and early postoperative complications were lower in the mediastinoscope group, and the number of resected mediastinal lymph nodes was greater. The results of this small study indicate that video mediastinoscope-assisted esophagolysis with trans-hiatal esophagectomy is safe and may reduce morbidity and mortality in ESCC patients [30]. Another risk of esophagectomy is complications from esophageal reconstruction. Tissue engineering using artificial scaffolding as an alternative technique for esophageal defect repair is under investigation in animal models. This approach improved survival, but graft stenosis and lack of motility were problematic and may limit use in humans [31].

The ideal scope of LN excision is debated. A number of comparative studies of three- versus two-field nodal dissection have been performed but with inconsistent results. Some have shown higher postoperative morbidity and mortality with more extensive lymphadenectomy, while others have shown lower LNM rate and improved long-term survival. These studies have largely discussed ESCC and EAC homogeneously despite current evidence suggesting a lower rate of LNM in ESCC. A meta-analysis by Wang et al. included 8 studies of 1676 patients with ESCC. Three-field lymphadenectomy yielded more total excised (not necessarily malignant) LN but also more blood loss and higher rate of the anastomotic fistula. There were no significant differences regarding number of malignant lymph nodes, OS, operation time, recurrent laryngeal nerve injury, pneumonia, chylothorax, anastomotic stenosis, ileus, cervical nodal recurrence, or hospital mortality. This indicates two-field lymphadenectomy may be preferred over three-field lymphadenectomy for use in ESCC, although further studies are needed [32].

## Combined modality therapy including surgery

The multicenter, phase III NEOCRTEC5010 trial evaluated outcomes after neoadjuvant CRT followed by surgery compared with surgery alone for locally advanced ESCC. Four hundred eleven patients were randomly assigned in a 1:1 ratio received neoadjuvant CRT (2 cycles of vinorelbine/cisplatin + concurrent RT – 40.0 Gy in 20 fractions) plus surgery (CRT + S) or surgery alone (S). At a median follow-up of 51.9 months, 62 patients (33.7%) in the CRT + S group versus 104 patients (45.8%) in the S group experienced recurrences (*P* = 0.013). The CRT + S group had significantly better loco-regional failure-free survival (*P* = 0.012) and lower distant metastasis-free survival (*P* = 0.028) than the S group. Recurrences occurred earlier in the S group (*P* = 0.053) and late relapses were more frequent in the CRT + S group (*P* = 0.029). The neoadjuvant CRT regimen was associated with significantly reduced loco-regional and distant recurrences compared with surgery alone, lending additional support to CRT plus surgery versus surgery alone for the treatment of locally advanced ESCC [33].

## Choice of CRT regimen

Combined modality therapy utilizing CRT with or without surgery for T2 or greater disease is standard of care [21]. Combinations of a platinum with a fluoropyrimidine or paclitaxel and radiotherapy are the norm, with the CROSS trial showing a greater benefit in ESCC versus EAC [34]. Induction chemotherapy is another option for patients undergoing surgery. There are also a number chemotherapy regimens used in this setting. Several recent studies evaluated both approaches.

A multicenter, randomized phase II trial compared concurrent CRT using either S-1 or S-1 and cisplatin in patients with inoperable ESCC. One hundred five patients were randomized and 89 were evaluable. Endoscopic complete response rate (46.9% in S-1 group versus 52.5% in S-1/cisplatin; similar to previous reports from other studies), OS (26 months versus not reached), and PFS (progression-free survival; 20 versus 21 months) were not significantly different after a mean follow-up time of 23 months. S-1 had less gastrointestinal and hematologic toxicities. A phase III trial is needed to confirm these findings [35].

Liu et al. investigated induction chemotherapy with docetaxel and cisplatin followed by definitive CRT versus CRT alone in patients with thoracic ESCC who were not surgical candidates. The CRT regimen was docetaxel/cisplatin and 60.0 Gy RT in 28 fractions. Response rate was 64.5% with induction chemotherapy versus 61.8% in the CRT alone group (*P* = 0.152). The 3-year survival rate was similar (41.8% versus 38.1%; *P* = 0.584; hazard ratio, 0.88; 95% CI, 0.54–1.41). Grade 3–5 AEs were similar. Given that induction chemotherapy did not improve RR or OS, different induction chemotherapy regimens or patient subgroups may need to be studied [36].

## Radiation therapy approach

Recent studies evaluated types of radiation, radiation resistance, and risk of recurrence following treatment with various RT modalities. Types of radiation studied include conventional field irradiation (CFI), elective nodal irradiation (ENI), involved-field irradiation (IFI), simultaneous integrated boost technique (SIBT), or intensity modulated radiotherapy (IMRT). Lyu et al. compared the effects of ENI versus IFI on long-term survival in stage II-III thoracic ESCC. Two hundred twenty-eight patients in China were enrolled. Chemotherapy was given concurrently with and following radiotherapy. Chemotherapy regimen was docetaxel and cisplatin. OS was 32.5 months with ENI versus 34.9 with IFI. After 5 years, OS was 29.8% with ENI and 30.7% with IFI. PFS was similarly insignificant. Significant decreases were seen in treatment-related esophagitis and pneumonitis with IFI versus ENI. The authors suggest IFI may be superior to ENI in treatment of stage II–III thoracic ESCC due to improved rates of adverse effects, with a similar effect on long-term survival [37]. In contrast, Xie et al. compared CRT with ENI versus CFI in 352 patients with locally advanced ESCC. CRT with paclitaxel/cisplatin was combined with 60 Gy of RT over 30 fractions. ENI significantly improved OS compared with CFI (median, 38.5 versus 22.6 months; HR, 0.74; *P* = 0.018) [38]. These trials taken together highlight the importance of individualized radiation plans based on cancer stage and tumor location.

Zhang et al. evaluated long-term survival and late adverse events (AEs) with radiation dose escalation by SIBT for locally advanced thoracic ESCC in an effort to better delineate the optimal radiation dose in CRT. Patients received escalating doses of RT at 4 levels (up to 70 Gy) concurrent with 2 cycles of cisplatin/fluorouracil followed by 2 additional cycles of chemotherapy. The 3-year OS rate was 57.6% with a PFS rate of 41%. There was favorable survival and tolerability with 63 Gy/50.4 Gy (boosting gross tumor to 63 Gy while 50.4 Gy is delivered to subclinical disease) in 28 fractions. Maximal dose to the esophagus was a significant predictor of SAEs [39]. Similarly, Ristau et al. studied SIBT in definitive RT. 70% of 101 total patients had ESCC. Definitive CRT or IMRT alone was given. CRT regimens involved IMRT and either cisplatin/5-FU, FOLFOX, or cetuximab. 58.8 Gy to the gross tumor and LN metastases and 50.4 Gy in 28 fraction to the primary tumor and elective LN was used. With a median follow-up of 17 months for the entire cohort, OS was 63.9% at 1 year and 37.6% at 3 years. OS, PFS, and local control rate (LCR) were at 63.9%, 53.9% and 59.9% after 1 year and 37.6%, 34.5%, and 36.1% after 3 years. Sixteen patients (15.8%) developed loco-regional recurrence within the RT field. 47.5% of patients experienced at least 1 grade III toxicity (mostly dysphagia) during RT. The use of concurrent chemotherapy was strongly associated with longer OS, PFS, and LCR. The authors concluded IMRT use in definitive CRT/RT is safe and feasible, with acceptable survival rates and moderate toxicity. Prospective studies are needed [40].

## Immunotherapy and targeted therapy

The landscape of immunotherapy is rapidly evolving, and at the forefront of new treatments for ESCC is immunotherapy and other targeted agents [41]. The CheckMate 577 trial involved 794 patients with resected stage II or III esophageal or GE junction cancer (30% ESCC) who had pathologic residual disease after trimodality therapy. Patients were randomized to receive either nivolumab or placebo. After a median follow-up of 24.4 months, disease-free survival was significantly longer in the nivolumab group, at 22.4 months (95% CI, 16.6–34.0) compared with 11 months (95% CI, 8.3–14.3) in the placebo group. The increased survival did not depend on programmed cell death ligand-1 (PD-L1) status. Health-related QOL was not adversely affected [42]. The US Food and Drug Administration (FDA) has now approved nivolumab for this use.

In a major leap forward, nivolumab was also approved by the US FDA in June of 2020 for the treatment of patients with unresectable advanced, recurrent, or metastatic ESCC after prior fluoropyrimidine- and platinum-based chemotherapy. This was based on results of the ATTRACTION-3 trial. Patients were randomized to receive either nivolumab or docetaxel/paclitaxel. Patients in the nivolumab group showed significantly greater OS of 10.9 months compared with 8.4 months in the chemotherapy group (hazard ratio = 0.77; 95% confidence interval: 0.62–0.96; *p* = .0189). In addition to demonstrating an overall survival benefit, the nivolumab group experienced less treatment-emergent or serious AE. Of note, the use of nivolumab in patients with ESCC was associated with a higher risk of esophageal fistula and pneumonitis than when used in patients with other cancer types [43].

Additional targeted treatments for ESCC have not completed phase III trials, but some show promising results in phase II clinical trials (see Table 2). These include the immune checkpoint inhibitors targeting the programmed cell death 1 (PD-1) receptors — nivolumab, pembrolizumab, and camrelizumab — the cyclin-dependent kinase 4/6 inhibitor palbociclib, and several tyrosine kinase inhibitors (apatinib, afatinib, anlotinib, icotinib, and erlotinib). In KEYNOTE-181, for example, pembrolizumab versus chemotherapy (investigator’s choice of paclitaxel, docetaxel, or irinotecan) as second-line treatment for advanced, unresectable esophageal cancer was evaluated. Median OS in ESCC was prolonged with pembrolizumab (8.2 months versus 7.1 months; HR, 0.78 [95% CI, 0.63 to 0.96]; *P* = .0095) in patients with PD-L1 CPS (combined positive score) ≥ 10 with fewer treatment-related AE (18.2% versus 40.9% experienced grade 3–5 AE). The results for locally advanced and unresectable ESCC were similarly favorable [44].

**Table 2:** Overview of recent clinical trials involving immunotherapy and targeted therapy for ESCC

Name/authors	Trial number	Tumor type	Setting (line)	Phase	Treatment aims	Key findings
CheckMate 577 [42]	NCT02743494	Resected stage II or III esophageal or GE junction cancer (30% ESCC)	Adjuvant, after neoadjuvant XRT + surgery with residual pathological disease	III	Cohort 1: nivolumab Cohort 2: placebo	DFS significantly longer with nivolumab regardless of PD-L1 CPS score (median 22.4 vs. 11 months; *p* < 0.001); OS data in progress
ATTRACTION-3 [43]	NCT02569242	Unresectable advanced, recurrent, or metastatic ESCC	Second-line	III	Cohort 1: nivolumab Cohort 2: chemotherapy (docetaxel or paclitaxel)	OS significantly longer with nivolumab (median 10.9 vs. 8.4 months; *p* = 0.019) with less AE
KEYNOTE-181 [44]	NCT02564263	Advanced, unresectable esophageal cancer (63% ESCC)	Second-line	III	Cohort 1: pembrolizumab Cohort 2: chemotherapy (investigator’s choice)	OS significantly longer with pembrolizumab when PD-L1 CPS score ≥ 10 (median 9.3 vs. 6.7 months; *p* = 0.0074) and with fewer treatment-related AE
KEYNOTE-590 [45]	NCT03189719	Locally advanced, unresectable, or metastatic esophageal cancer (73% SCC)	First-line	III	Cohort 1: pembrolizumab + chemotherapy (cisplatin + 5-FU) Cohort 2: placebo + chemotherapy (cisplatin + 5-FU)	OS in ESCC significantly longer with pembrolizumab irrespective of PD-L1 CPS score (median 12.6 vs. 9.8 months; *p* = 0.0006); PFS also significantly longer (6.3 vs. 5.8 months; *p* < 0.0001)
Zhang et al. [46]	NCT03603756	Unresectable locally advanced or recurrent/metastatic ESCC	First-line	II	Cohort 1: camrelizumab, apatinib, and chemotherapy (paclitaxel and nedaplatin)	Median OS of 19.43 months, median PFS of 6.85 months, and mild AE
ESO-Shanghai 11 [47]	NCT03274011	Chemotherapy-refractory ESCC	Second-line	II	Cohort 1: apatinib	Median OS of 5.8 months, median PFS of 3.8 months, and mild AE in patients with controlled primary tumors without major vessel invasion
Hong et al. [48]	NCT02353936	Recurrent/metastatic ESCC	Second-line	II	Cohort 1: afatinib	Median OS of 6.3 months, median PFS of 3.4 months, and mild AE
ALTER1102 [49]	NCT02649361	Recurrent/metastatic ESCC	Second-line	II	Cohort 1: anlotinib Cohort 2: placebo	Median PFS significantly improved with anlotinib (median 3.02 vs. 1.41 months; *p* < 0.001), and tolerable AE
Xie et al. [38]	NCT00686114	Locally advanced, inoperable ESCC	First-line	III	Cohort 1: erlotinib + CRT (paclitaxel/carboplatin) Cohort 2: CRT (paclitaxel/carboplatin)	OS significantly longer with erlotinib (median 39.4 vs. 27.4 months; *p* = 0.025)
Luo et al. [50]	NCT02375581	Unresectable ESCC in older patients	First-line	II	Cohort 1: icotinib + RT Cohort 2: RT	OS significantly longer with icotinib (median 24 vs. 16.3 months; *p* = 0.008) with no difference in grade 3–4 AE
Karasic et al. [51]	NCT01037790	Locally advanced or metastatic esophageal or gastric cancer (24% ESCC)	Second-line	II	Cohort 1: Palbociclib	No objective response, median OS 3 months, median PFS 1.8 months, and grade 3–4 hematologic AE; minimal clinical activity in this population
Shiozaki et al. [52]	jRCTs051190076	Stage II–III ESCC	First-line	I–II	Cohort 1: tranilast + chemotherapy (cisplatin + 5-FU)	No data yet

*AE*, adverse events; *ESCC*, esophageal squamous cell carcinoma; *GE*, gastroesophageal; *OS*, overall survival; *PFS*, progression-free survival

## Biomarkers

Many trials include an evaluation of predictive biomarkers. The most commonly used biomarker for prediction of activity of PD-1 blocking drugs is the PD-L1 CPS. In KEYNOTE-181 pembrolizumab improved OS versus chemotherapy as second-line treatment of advanced, unresectable esophageal cancer only when PD-L1 CPS was ≥ 10 [44]. In contrast, in KEYNOTE-590 pembrolizumab plus chemotherapy versus chemotherapy alone improved OS and PFS in ESCC regardless of CPS score [45]. Correlation of treatment response with biomarker presence is not limited to monoclonal antibody based therapies. Studies are demonstrating this with other types of targeted therapies as well (see Table 3).

**Table 3:** Overview of biomarkers recently studied in ESCC

Name/authors	Biomarker studied	Study type	Tumor type	Key findings
KEYNOTE-181 [44]	PD-1	Phase III clinical trial	Advanced, unresectable esophageal cancer (63% ESCC)	OS was significantly longer with pembrolizumab when PD-L1 CPS score ≥ 10 and with fewer treatment-related AE.
KEYNOTE-590 [45]	PD-1	Phase III clinical trial	Locally advanced, unresectable, or metastatic esophageal cancer (73% ESCC)	OS and PFS were significantly longer with pembrolizumab + chemotherapy (cisplatin + 5-FU) irrespective of PD-L1 CPS score, vs. placebo + the same chemotherapy regimen.
Egebjerg et al. [53]	HER2	Systematic review and meta-analysis involving 18 studies	ESCC, unspecified stage	Estimated prevalence of HER2 positivity was 10% by ISH and 8.6% by IHC followed by ISH.
Zhang et al. [54]	ANO1	Systematic review and meta-analysis involving one relevant study	ESCC, unspecified stage	ANO1 overexpression was significantly associated with poor OS.
Zhao et al. [55]	Oct4	Systematic review and meta-analysis involving two relevant studies	ESCC, unspecified stage	Oct4 overexpression was significantly associated with poor OS.
Ishibashi et al. [56]	PLR	Systematic review and meta-analysis involving 10 retrospective reviews	ESCC, various stages	High PLR was significantly associated with poor OS.
Ishibashi et al. [56]	Platelet count	Systematic review and meta-analysis involving four retrospective reviews	ESCC, various stages	Platelet count was a non-independent prognostic factor for OS.
Ishibashi et al. [56]	PDW	Systematic review and meta-analysis involving three retrospective reviews	ESCC, various stages	PDW was a non-independent prognostic factor for OS.
Ishibashi et al. [56]	MPV	Systematic review and meta-analysis involving three retrospective reviews	ESCC, various stages	MPV was a non-independent prognostic factor for OS.
Liu et al. [57]	ctDNA	Single-center cohort study involving 23 patients	Resectable ESCC, various stages	ctDNA detection after tumor resection was significantly associated shorter OS and higher relapse rates compared to postoperative ctDNA-negative status.
Jia et al. [58]	ctDNA	Single-center cohort study involving 25 patients	Locally advanced ESCC	ctDNA positivity in post-radiation sample was significantly associated with a lower OS and PFS compared to patients with ctDNA-negative post-radiation samples.
Ujiie et al. [59]	ctDNA	Single-center cohort study involving 30 patients	ESCC, primarily stages II–III	Following neoadjuvant 5-FU and cisplatin, early recurrence was associated with a significantly higher number of total circulating tumor cells before chemotherapy and a significantly higher number of mesenchymal-type circulating tumor cells after chemotherapy when compared to the group without recurrence.

*AE*, adverse events; *ANO1*, anoctamin-1; *ctDNA*, circulating tumor DNA (peripheral blood); *ESCC*, esophageal squamous cell carcinoma; *HER2*, human epidermal growth factor receptor-2; *IHC*, immunohistochemistry; *ISH*, in situ hybridization; *MPV*, mean platelet volume; *Oct4*, octamer-binding transcription factor; *OS*, overall survival; *PD-1*, programmed cell death protein-1; *PD-L1 CPS*, programmed cell death ligand-1 combined positive score; *PDW*, platelet distribution width; *PFS*, progression-free survival; *PLR*, platelet-to-lymphocyte ratio

HER2 overexpression is a predictive marker in EAC. Regarding expression in ESCC, a systematic review and meta-analysis by Egebjerg et al. showed the estimated prevalence of HER2 positivity in ESCC to be 10% as assessed by in situ hybridization (ISH) and 8.6% as assessed by initial immunohistochemistry (IHC) followed by ISH, indicating potential for anti-HER2 targeted therapy in ESCC, for which there are studies underway [53].

A few recent studies regarding the genomics of ESCC aim to better elucidate shared genetic susceptibility between ESCC and other cancer types the genetic basis of radiation therapy resistance, and possible clinical utility of genes as prognostic factors [60]. Zhou et al. identified common gene signatures and functional pathways shared between the most common double primary aerodigestive tumors with a poor prognosis — hypopharyngeal and esophageal SCC, thus contributing to our understanding of the molecular mechanisms of ESCC carcinogenesis and progression. Forty-three differentially expressed genes were identified, 25 upregulated and 18 downregulated genes. Survival analysis showed SERPINE1 and SPP1 to be related to poor prognosis in patients with ESCC and hypopharyngeal SCC [61]. Yang et al. did whole-exome sequencing to evaluate the genetic basis of RT resistance in ESCC. This was performed on 42 patient samples obtained throughout the course of RT. Single-cell whole-exome sequencing was done on 147 cells from 2 patients. Significant allelic changes occurred during irradiation, including modulation of 42 putative radio-responsive genes including MAML3, CDKN2A, NFE2L2, GAS2L2, OBSCN, and TP53. Mutations in ten genes predicted improved prognosis, while those in 18 genes were associated with significantly shortened time to loco-regional recurrence [62].

One retrospective study aimed to develop a prognostic model for clinical stratification of stage IB/IIA ESCC to screen out subgroups with poor prognosis prior to undergoing esophagectomy. One hundred fifty-three patients with a history of IB/IIAA ESCC who had undergone esophagectomy were retrospectively analyzed, including the analysis of PD-L1 expression in their paraffin tissue. Univariable and multivariable analyses identified pTNM stage, number of lymph nodes, and PD-L1 expression as independent predictors of OS. All were adversely related with OS. These components comprise their three-factor prognostic score, which demonstrated an advantage over purely TNM staging in the ability to identify high-risk patients (C-index = 0.774 versus C-index = 0.570, *P* < 0.001) [63].

## Palliative treatment

Palliative treatment is individualized with the goal of maximizing QOL. Persistent dysphagia can be palliated with self-expanding metal stent (SEMS) insertion, but recurrent dysphagia often requires repeat intervention. A multicenter, open-label, randomized, controlled phase III trial evaluated the efficacy of palliative adjuvant external beam radiotherapy (EBRT) after esophageal cancer stenting compared with stenting alone in preventing dysphagia deterioration and reducing healthcare service use (clinician visits, nursing home visits, hospice care, and medication use) in 220 patients with esophageal or GE junction carcinoma after SEMS insertion. One-third of patients had ESCC versus EAC. In the EBRT group, RT was given within 4 weeks of stent insertion, preferably 2 weeks. No additional benefit was provided by palliative radiotherapy given concurrently with SEMS insertion [64].

## References

[1] Fitzmaurice C, Dicker D, Pain A (2015). The global burden of cancer 2013. JAMA Oncol.

[2] Sung H, Ferlay J, Siegel RL (2021). Global cancer statistics 2020: GLOBOCAN estimates of incidence and mortality worldwide for 36 cancers in 185 countries. CA Cancer J Clin.

[3] Arnold M, Soerjomataram I, Ferlay J, Forman D (2015). Global incidence of oesophageal cancer by histological subtype in 2012. Gut.

[4] Bray F, Ferlay J, Soerjomataram I, Siegel RL, Torre LA, Jemal A (2018). Global cancer statistics 2018: GLOBOCAN estimates of incidence and mortality worldwide for 36 cancers in 185 countries. CA Cancer J Clin.

[5] Tran GD, Sun XD, Abnet CC (2005). Prospective study of risk factors for esophageal and gastric cancers in the Linxian general population trial cohort in China. Int J Cancer.

[6] Gholipour C, Shalchi RA, Abbasi M (2008). A histopathological study of esophageal cancer on the western side of the Caspian littoral from 1994 to 2003. Dis Esophagus.

[7] Thrift AP, Shaheen NJ, Gammon MD, et al. Obesity and risk of esophageal adenocarcinoma and Barrett’s esophagus: a Mendelian randomization study. J Natl Cancer Inst. 2014;106(11). 10.1093/jnci/dju252. 10.1093/jnci/dju252 PMC4200028 25269698

[8] He L, Jin-Hu F, You-Lin Q, He L, Jin-Hu F, You-Lin Q (2017). Epidemiology, etiology, and prevention of esophageal squamous cell carcinoma in China. Cancer Biol Med.

[9] Hölscher AH, Bollschweiler E, Schneider PM, Siewert JR (1995). Prognosis of early esophageal cancer. Comparison between adeno-and squamous cell carcinoma. Cancer..

[10] Paraf F, Fléjou JF, Pignon JP, Fékété F, Potet F (1995). Surgical pathology of adenocarcinoma arising in Barrettʼs esophagus analysis of 67 cases. Am J Surg Pathol.

[11] Cavallin F, Scarpa M, Cagol M (2018). Esophageal cancer clinical presentation: trends in the last 3 decades in a large Italian series. Ann Surg.

[12] Hanaoka N, Ishihara R, Motoori M (2018). Endoscopic balloon dilation followed by intralesional steroid injection for anastomotic strictures after esophagectomy: a randomized controlled trial. Am J Gastroenterol.

[13] Luo LN, He LJ, Gao XY (2016). Evaluation of preoperative staging for esophageal squamous cell carcinoma. WJG..

[14] Dawsey SM, Wang GQ, Weinstein WM (1993). Squamous dysplasia and early esophageal cancer in the Linxian region of China: distinctive endoscopic lesions. Gastroenterology..

[15] van Vliet EPM, Heijenbrok-Kal MH, Hunink MGM, Kuipers EJ, Siersema PD (2008). Staging investigations for oesophageal cancer: a meta-analysis. Br J Cancer.

[16] Rice TW, Kelsen D, Blackstone EH, Amin MB, Edge SB, Greene FL (2017). Esophagus and esophagogastric junction. AJCC cancer staging manual.

[17] He H, Chen N, Hou Y (2020). Trends in the incidence and survival of patients with esophageal cancer: a SEER database analysis. Thorac Cancer.

[18] Petrelli F, De Santi G, Rampulla V (2021). Human papillomavirus (HPV) types 16 and 18 infection and esophageal squamous cell carcinoma: a systematic review and meta-analysis. J Cancer Res Clin Oncol.

[19] Lee SL, Yadav P, Starekova J (2021). Diagnostic performance of MRI for esophageal carcinoma: a systematic review and meta-analysis. Radiology..

[20] Lordick F, Mariette C, Haustermans K, Obermannová R, Arnold D (2016). Oesophageal cancer: ESMO clinical practice guidelines for diagnosis, treatment and follow-up. Ann Oncol.

[21] McNamara MJ, Adelstein DJ (2012). Current developments in the management of locally advanced esophageal cancer. Curr Oncol Rep.

[22] Miyata H, Yamasaki M, Kurokawa Y (2011). Multimodal treatment for resectable esophageal cancer. Gen Thorac Cardiovasc Surg.

[23] Wang H, Zeng X, Bai S (2020). The safety and efficacy of endoscopic submucosal dissection for treating early oesophageal carcinoma: a meta-analysis. Annals.

[24] Han C, Sun Y (2021). Efficacy and safety of endoscopic submucosal dissection versus endoscopic mucosal resection for superficial esophageal carcinoma: a systematic review and meta-analysis. Dis Esophagus.

[25] Zhang Y, Liu L, Wang Q (2020). Endoscopic submucosal dissection with additional radiotherapy in the treatment of T1a esophageal squamous cell cancer: randomized controlled Trial. Endoscopy..

[26] Xu W, Bo LX, Bao LS, Hao YZ, Tong Q (2020). Prediction of lymph node metastasis in superficial esophageal squamous cell carcinoma in Asia: a systematic review and meta-analysis. Dis Esophagus.

[27] Jiang KY, Huang H, Chen WY (2021). Risk factors for lymph node metastasis in T1 esophageal squamous cell carcinoma: a systematic review and meta-analysis. WJG..

[28] Tachimori Y (2017). Pattern of lymph node metastases of squamous cell esophageal cancer based on the anatomical lymphatic drainage system: efficacy of lymph node dissection according to tumor location. J Thorac Dis.

[29] Wilk BJ, Eyck BM, Doukas M (2020). Residual disease after neoadjuvant chemoradiotherapy for oesophageal cancer: locations undetected by endoscopic biopsies in the preSANO trial. Br J Surg.

[30] Rezaei R, Masuom SHF, Soroush N, Zehi V (2021). Comparison of transhiatal esophagectomy using a mediastinoscope with transhiatal esophagectomy by the classic method. Asian Cardiovasc Thorac Ann.

[31] Schizas D, Frountzas M, Sgouromallis E (2021). Esophageal defect repair by artificial scaffolds: a systematic review of experimental studies and proportional meta-analysis. Dis Esophagus.

[32] Wang J, Yang Y, Shafiulla Shaik M (2020). Three-field versus two-field lymphadenectomy for esophageal squamous cell carcinoma: a meta-analysis. J Surg Res.

[33] Liu S, Wen J, Yang H (2020). Recurrence patterns after neoadjuvant chemoradiotherapy compared with surgery alone in oesophageal squamous cell carcinoma: results from the multicenter phase III trial NEOCRTEC5010. Eur J Cancer.

[34] van Hagen P, Hulshof MCCM, van Lanschot JJB (2012). Preoperative chemoradiotherapy for esophageal or junctional cancer. N Engl J Med.

[35] Zhao Z, Wen Y, Liao D, et al. Single-agent versus double-agent chemotherapy in concurrent chemoradiotherapy for esophageal squamous cell carcinoma: prospective, randomized, multicenter phase II clinical trial. Oncologist. 2020;25(12). 10.1634/theoncologist.2020-0808. 10.1634/theoncologist.2020-0808 PMC8108049 32864805

[36] Liu S, Luo L, Zhao L (2021). Induction chemotherapy followed by definitive chemoradiotherapy versus chemoradiotherapy alone in esophageal squamous cell carcinoma: a randomized phase II trial. Nat Commun.

[37] Lyu J, Yisikandaer A, Li T (2020). Comparison between the effects of elective nodal irradiation and involved-field irradiation on long-term survival in thoracic esophageal squamous cell carcinoma patients: a prospective, multicenter, randomized, controlled study in China. Cancer Med.

[38] Xie C, Jing Z, Luo H (2020). Chemoradiotherapy with extended nodal irradiation and/or erlotinib in locally advanced oesophageal squamous cell cancer: long-term update of a randomised phase 3 trial. Br J Cancer.

[39] Zhang Y, Feng W, Gao LT (2021). Long-term follow-up of a phase I/II trial of radiation dose escalation by simultaneous integrated boost for locally advanced esophageal squamous cell carcinoma. Radiother Oncol.

[40] Ristau J, Thiel M, Katayama S (2021). Simultaneous integrated boost concepts in definitive radiation therapy for esophageal cancer: outcomes and toxicity. Radiat Oncol.

[41] Puhr HC, Preusser M, Ilhan-Mutlu A (2021). Immunotherapy for esophageal cancers: what is practice changing in 2021?. Cancers..

[42.•] Kelly RJ, Ajani JA, Kuzdzal J (2021). Adjuvant nivolumab in resected esophageal or gastroesophageal junction cancer. N Engl J Med.

[43.•] Kato K, Cho BC, Takahashi M (2019). Nivolumab versus chemotherapy in patients with advanced oesophageal squamous cell carcinoma refractory or intolerant to previous chemotherapy (ATTRACTION-3): a multicentre, randomised, open-label, phase 3 trial. Lancet Oncol.

[44.•] Kojima T, Shah MA, Muro K (2020). Randomized phase III KEYNOTE-181 study of pembrolizumab versus chemotherapy in advanced esophageal cancer. JCO.

[45] Sun JM, Shen L, Shah MA (2021). Pembrolizumab plus chemotherapy versus chemotherapy alone for first-line treatment of advanced oesophageal cancer (KEYNOTE-590): a randomised, placebo-controlled, phase 3 study. Lancet.

[46] Zhang B, Qi L, Wang X (2020). Phase II clinical trial using camrelizumab combined with apatinib and chemotherapy as the first-line treatment of advanced esophageal squamous cell carcinoma. Cancer Commun.

[47] Chu L, Chen Y, Liu Q, et al. A phase II study of apatinib in patients with chemotherapy-refractory esophageal squamous cell carcinoma (ESO-Shanghai 11). Oncologist. 2021;26(6). 10.1002/onco.13668. 10.1002/onco.13668 PMC8176978 33393167

[48] Hong MH, Heo SG, Lee Y (2020). Phase 2 study of afatinib among patients with recurrent and/or metastatic esophageal squamous cell carcinoma. Cancer..

[49] Huang J, Xiao J, Fang W (2021). Anlotinib for previously treated advanced or metastatic esophageal squamous cell carcinoma: a double-blind randomized phase 2 trial. Cancer Med.

[50] Luo H, Jiang W, Ma L (2020). Icotinib with concurrent radiotherapy vs radiotherapy alone in older adults with unresectable esophageal squamous cell carcinoma: a phase II randomized clinical trial. JAMA Netw Open.

[51] Karasic TB, O’Hara MH, Teitelbaum UR, et al. Phase II trial of palbociclib in patients with advanced esophageal or gastric cancer. Oncologist. 2020;25(12). 10.1634/theoncologist.2020-0681. 10.1634/theoncologist.2020-0681 PMC8108046 32692450

[52] Shiozaki A, Kudou M, Fujiwara H (2020). Clinical safety and efficacy of neoadjuvant combination chemotherapy of tranilast in advanced esophageal squamous cell carcinoma: phase I/II study (TNAC). Medicine..

[53] Egebjerg K, Garbyal RS, Hasselby JP, Baeksgaard L, Mau-Sørensen M (2021). Prevalence of HER2 overexpression and amplification in squamous cell carcinoma of the esophagus: a systematic review and meta-analysis. Crit Rev Oncol Hematol.

[54] Zhang C, Li H, Gao J, Cui X, Yang S, Liu Z (2021). Prognostic significance of ANO1 expression in cancers. Medicine..

[55] Zhao X, Lu H, Sun Y, Liu L, Wang H (2020). Prognostic value of octamer binding transcription factor 4 for patients with solid tumors: a meta-analysis. Medicine..

[56] Ishibashi Y, Tsujimoto H, Sugasawa H (2021). Prognostic value of platelet-related measures for overall survival in esophageal squamous cell carcinoma: a systematic review and meta-analysis. Crit Rev Oncol Hematol.

[57] Liu T, Yao Q, Jin H (2021). Plasma circulating tumor DNA sequencing predicts minimal residual disease in resectable esophageal squamous cell carcinoma. Front Oncol.

[58] Jia R, Zhao CH, Li PS (2020). Post-radiation circulating tumor DNA as a prognostic factor in locally advanced esophageal squamous cell carcinoma. Oncol Lett.

[59] Ujiie D, Matsumoto T, Endo E (2021). Circulating tumor cells after neoadjuvant chemotherapy are related with recurrence in esophageal squamous cell carcinoma. Esophagus..

[60] Lesseur C, Ferreiro-Iglesias A, McKay JD (2021). Genome-wide association meta-analysis identifies pleiotropic risk loci for aerodigestive squamous cell cancers. Chanock SJ, ed. PLoS Genet.

[61] Zhou R, Liu D, Zhu J, Zhang T (2020). Common gene signatures and key pathways in hypopharyngeal and esophageal squamous cell carcinoma: evidence from bioinformatic analysis. Medicine..

[62] Yang L, Zhang X, MacKay M (2020). Identification of radioresponsive genes in esophageal cancer from longitudinal and single cell exome sequencing. Int J Radiat Oncol Biol Phys.

[63] Wu LL, Ma QL, Huang W (2021). A prognostic model for stratification of stage IB/IIA esophageal squamous cell carcinoma: a retrospective study. BMC Gastroenterol.

[64] Adamson D, Byrne A, Porter C (2021). Palliative radiotherapy after oesophageal cancer stenting (ROCS): a multicentre, open-label, phase 3 randomised controlled trial. Lancet Gastroenterol Hepatol.

